# An *In Vitro* Approach to Evaluate the Impact of Autolysis and Formalin Fixation on the Detection of Canine Distemper Virus and Innate Immune Response Antigens

**DOI:** 10.3390/v17121575

**Published:** 2025-12-02

**Authors:** Hannah Gerhards, Karl Rohn, Christina Puff, Wolfgang Baumgärtner

**Affiliations:** 1 Department of Pathology, University of Veterinary Medicine Hannover, Foundation, 30559 Hannover, Germany; hannah.gerhards@tiho-hannover.de (H.G.); christina.puff@tiho-hannover.de (C.P.); 2 Department of Biometry, Epidemiology and Data Management, University of Veterinary Medicine Hannover, Foundation, 30559 Hannover, Germany; karl.rohn@tiho-hannover.de

**Keywords:** autolysis, canine distemper virus, formalin fixation, immunohistochemistry, interferon-stimulated genes, virus-sensing tools

## Abstract

Viral infections in humans and animals are increasing, and retrospective studies using formalin-fixed, paraffin-embedded (FFPE) samples reveal recurring outbreaks over past decades. However, the impact of pre-analytical factors like fixation and autolysis on immunohistochemistry (IHC) remains insufficiently understood. To examine how autolysis, fixation duration (6–72 h) and formalin concentration (2.5–25%) influence histology and IHC of canine distemper virus (CDV, *Morbillivirus canis*), interferon-β (IFN-β), and selected IFN-stimulated genes (ISGs), the study was conducted using an *in vitro* model based on persistently CDV-infected and non-infected DH82 cells (canine histiocytic sarcoma cell line). Autolysis led to a progressive loss of cell morphology, whereas formalin fixation had minimal impact. CDV nucleoprotein, ISG15, and myxovirus resistance protein (Mx) showed stable immunohistochemical signals across all fixation conditions and remained detectable after prolonged autolysis. CDV infection upregulated ISG15 and Mx. In contrast, IFN-β and phosphorylated protein kinase R (pPKR) exhibited variable staining and did not distinguish infected from non-infected samples. Overall, autolysis had a stronger negative impact on IHC signal quality than fixation parameters. Despite the limitations of the *in vitro* model, the robustness of CDV, ISG15, and Mx under suboptimal conditions highlights their potential utility as virus-sensing markers in FFPE material.

## 1. Introduction

Viral infectious diseases represent a major threat to both animal and human health and are frequently associated with substantial economic consequences. In particular, zoonotic infections are increasingly responsible for global epidemics and pandemics [1,2,3]. Retrospective studies have further demonstrated that viral outbreaks have occurred repeatedly over past decades [4,5]. In diagnostic pathology, histopathology and immunohistochemistry (IHC) are essential tools for assessing tissue alterations and identifying pathogens [6,7]. While histopathology offers morphological evaluation, IHC allows the visualization of specific proteins within histological lesions. This technique supports tumor classification and pathogen detection and has become an essential tool in both human and veterinary diagnostics [6,8,9]. Successful immunohistochemical staining is dependent on the preservation of epitopes, which is generally achieved through chemical fixation, most commonly with 10% neutral buffered formalin (NBF) [8]. Formalin has been the standard fixative in pathology for decades due to its ability to preserve tissues through the formation of inter- and intramolecular cross-links between proteins and nucleic acids [10,11,12]. These cross-links inhibit enzymatic degradation and stabilize the tissue microarchitecture [11,13]. Formalin fixation, followed by paraffin embedding, results in formalin-fixed, paraffin-embedded (FFPE) tissues, which are ideal for long-term storage and subsequent retrospective analysis [9,14,15]. However, the process of formalin fixation presents limitations. Formaldehyde, the active component in formalin, can alter protein conformation and mask epitopes, thereby reducing immunoreactivity [6,16,17]. Factors such as fixation time, formalin concentration, and tissue thickness can influence staining outcomes [12,13,18]. In particular, incomplete or prolonged fixation may result in artifacts, reduced antigen accessibility, and uneven staining patterns [12,13,19]. Although heat-induced epitope retrieval and enzymatic digestion can partially restore antigenicity, their effectiveness varies on the antibody and antigen involved [19,20,21]. In addition to fixation-related issues, autolysis accounts for another challenge in IHC. Autolysis starts shortly after death by adenosine triphosphate (ATP) depletion, leading to impaired membrane integrity, enzyme release, and cellular breakdown [22,23,24]. The degree of autolysis is tissue dependent and influenced by environmental conditions such as temperature, humidity, and pH [25]. Autolysis compromises both morphological and molecular integrity, resulting in poor tissue preservation, indistinct cellular boundaries, and degradation of immunoreactive proteins, particularly phosphorylated epitopes [12,26,27]. Consequently, autolysis not only hinders histological interpretation but also alters the results of immunohistochemical analyses [23,27,28]. Given the central role of IHC in diagnostics, it is crucial to understand the effects of these pre-analytical variables on the sensitivity and specificity of antigen detection.

In the context of infectious diseases and the detection of emerging and reemerging diseases in animals, archived FFPE tissues are often the only available source for retrospective studies [29,30]. A common issue with archived material for retrospective studies is the poor documentation of pre-analytical factors. In many cases, the time interval between the death of the animal and the collection of tissue during necropsy as well as formalin concentrations used for fixation are either unspecified or only vaguely reported [15]. Both variables may influence the sensitivity and reliability of retrospective analyses. FFPE-based virus diagnostics rely almost exclusively on virus-specific antibodies [31,32]. However, in cases of suspected viral cause but unknown etiology, broadly applicable bio-markers for the general detection of viral infections in FFPE tissues would be helpful. One strategy for early detection of viral outbreaks involves the use of antibodies directed against double-stranded ribonucleic acids (dsRNA). This replication intermediate is produced during viral infections and plays a critical role in triggering the host immune response [33]. Another promising approach lies in the use of host-derived markers that are upregulated during viral infection. Promising candidates for immunohistochemical detection are components of the type I interferon (IFN) signaling pathway, a central element of the host’s innate antiviral defense. Activation of this pathway is initiated by the recognition of dsRNA through pattern recognition receptors (PRRs) such as protein kinase R (PKR) [32,34,35,36,37,38,39,40]. Upon activation, the IFN-β signaling cascade induces the expression of IFN-stimulated genes (ISGs), which establish an antiviral state by inhibiting viral replication, promoting apoptosis of infected cells, and modulating immune responses [39,41,42]. Among the best-characterized ISGs are myxovirus resistance protein (Mx) and ISG15, both of which are upregulated during viral infections and have been shown to inhibit viral replication [37,43,44,45,46,47,48]. However, their expression can also be induced by non-viral stimuli, raising concerns about specificity [49]. Furthermore, many viruses have evolved mechanisms to evade innate immune sensing, complicating the interpretation of biomarker expression in FFPE tissue [36,40,50,51,52]. Finally, there are species-dependent expression patterns of type I IFN components [53]. Nonetheless, the IFN pathway remains a highly conserved and robust antiviral mechanism, and its components may be useful in identifying viral infections.

In this context, canine distemper virus (now *Morbillivirus canis*, CDV) represents an interesting model pathogen. CDV is a morbillivirus of the family *Paramyxoviridae* [54,55], and it infects a wide range of carnivores, including domestic dogs and numerous wild and endangered species [56]. CDV is neurotropic and capable of inducing a demyelinating leukoencephalitis [57,58,59]. Given the complexity of CDV pathology and its relevance in both clinical and ecological contexts, reliable immunohistochemical detection methods are of high importance. To investigate virus-induced immune responses in a controlled setting, the DH82 canine histiocytic sarcoma cell line is a valuable *in vitro* model. Originally derived from a disseminated canine histiocytic sarcoma, DH82 cells retain key macrophage functions and are susceptible to CDV infection, making them suitable for studying host–pathogen interactions [60,61,62]. Macrophages, a key element of the innate immune response, reside in all tissues, and DH82 cells express characteristic macrophage markers and cytokines [60,61]. However, it must be emphasized that the use of one single cell line and one virus lacks the complexity observed in tissue samples under real life conditions.

The aim of the present study is to evaluate the effects of the key pre-analytical variables autolysis, formalin fixation time, and formalin concentration on the immunohistochemical detectability of CDV and canine host innate immune markers. Particular attention is given to components of the type I IFN response. The selected experimental conditions are designed to reflect realistic scenarios encountered in daily necropsy situations, with the goal to identify robust biomarkers suitable for retrospective viral diagnostics in FFPE material, while acknowledging that translation to tissue environments is limited using an *in vitro* system only. However, certain factors that influence individual cells are difficult to analyze within the tissue microenvironment due to complex multi- and bidirectional crosstalk interactions. Single cell analyses in a reductionistic system can therefore provide important insights, although such study designs only partially mimic the complexity of the *in vivo* scenario. Ultimately, this work aims to contribute to the development of standardized protocols and improve the interpretability of IHC-based viral detection methods in veterinary pathology.

## 2. Materials and Methods

### 2.1. Cell Culture

DH82 cells represent a histiocytic sarcoma cell line derived from the bone marrow of a dog diagnosed with disseminated histiocytic sarcoma [62]. DH82 cells were obtained from the European Collection of Authenticated Cell Cultures (ECACC, No. 94062922). These cells express Fcγ receptors on their surface and exhibit phagocytic activity, reflecting key functional characteristics of macrophages [62]. DH82 cells were infected with the “Onderstepoort” (Ond) strain of CDV (GenBank No.: AF378705.1) at passage 104. Following serial passaging over several weeks, a stable infected cell population with approximately 100% CDV antigen positive cells (DH82 CDV-Ond p.i.) was established [63].

Infected and non-infected cells were cultivated in Minimum Essential Medium (MEM, Gibco, Thermo Fisher Scientific, Cat.-No. 11095080, Waltham, MA, USA) with addition of 10% fetal bovine serum (Capricorn Scientific GmbH, Cat.-No. FBS-DIA-12B, Ebsdorfergrund, Germany), 1% penicillin-streptomycin (P.0781, Sigma Aldrich, St. Louis, MO, USA), and 1% MEM non-essential amino acid solution (SIGMA Life Science, M7145, St. Louis, MO, USA) under standard conditions (37 °C, 5% CO_2_, water-saturated atmosphere). The medium was changed every three to four days and the cells were passaged in weekly intervals. Passages 8–13 of non-infected and passages 143–151 of DH82 CDV-Ond p.i. cells were used in the present study. For the preparation of cell pellets, the adherent cells of a 75 cm^2^ cell culture flask (Nunc, EasYFlask, Nunclon, Δ Surface, F7552, Sigma-Aldrich, St. Louis, MO, USA) with a confluent monolayer were gently detached from the bottom of the flask using a cell scraper (Sarstedt Inc, Cat-No. 83.1830, Newton, NC, USA). The obtained cell suspension was centrifuged (250× *g*, 4 °C, 10 min), and the supernatant was discarded. A volume of 200 µL of a cell suspension was stained with 400 µL trypan blue solution, and the total number of cells was determined using a hemocytometer. A volume of 15 million cells was washed twice with phosphate-buffered saline (PBS, containing 137 mM sodium chloride, 2.7 mM potassium chloride, and 12 mM total phosphate, pH 7.4) and subsequently centrifuged (250× *g*, 4 °C, 10 min). The resulting cell pellet was resuspended with 1 mL PBS, transferred to a 1.5 mL tube (Eppendorf SE, Cat.-No. 0030120086, Hamburg, Germany), and centrifuged again (300× *g*, 4 °C, 5 min). After the last centrifugation step, the supernatant was discarded.

**Figure 1:**
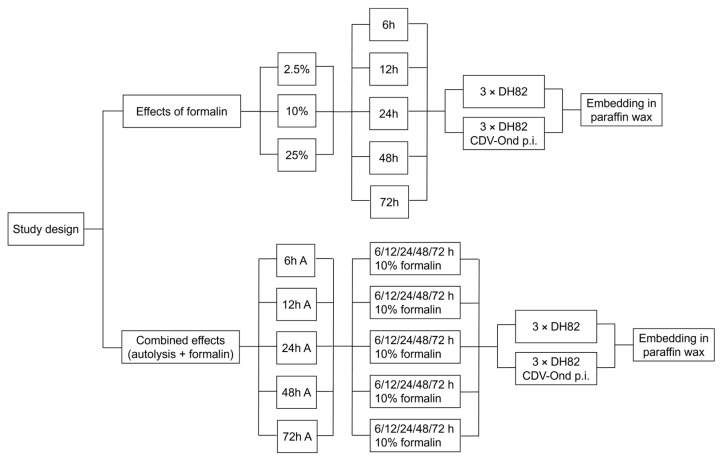
Two experimental approaches were performed to assess the effects of fixation and autolysis on histology and immunohistochemistry. In the first setup, persistently canine distemper virus (CDV; *Morbillivirus canis*) strain “Onderstepoort” infected and non-infected DH82 cell pellets were fixed in 2.5%, 10%, or 25% neutral buffered formalin (NBF) for 6, 12, 24, 48, or 72 h at room temperature, followed by paraffin embedding. In the second setup, analyzing the combined effects of autolysis and subsequent formalin fixation, pellets were first incubated in phosphate-buffered saline (PBS) at room temperature for 6, 12, 24, 48, or 72 h to simulate autolysis. After centrifugation and removal of the supernatant, pellets were fixed in 10% NBF for 6, 12, 24, 48, or 72 h before embedding in paraffin. All conditions were performed in triplicates for both CDV infected and non-infected cells. 2.5%/10%/25% formalin = solution of 1%/4%/10% formaldehyde in water; A = autolysis.

In the first experimental approach (Figure 1, outlined study design), the effects of formalin fixation on histological changes and immunohistochemical reaction patterns were systematically investigated. To simulate different fixative concentrations, 1 mL of 2.5%, 10%, and 25% NBF (solution of 1%, 4%, 10% formaldehyde in water, supplemented with mono- and diphasic sodium phosphate, pH 7.4) was added to the cell pellets. For investigation of the influence of the fixation time, cell pellets were fixed for 6, 12, 24, 48, and 72 h at room temperature (RT; 20 °C). Thereafter, cell pellets were embedded in paraffin wax. All conditions and time points were investigated using triplicates of non-infected and infected DH82 cells. In a second experimental setup (Figure 1 outlined study design), the combined effects of autolysis and subsequent formalin fixation were evaluated. To simulate autolysis three cell pellets of non-infected and infected cells were incubated in 1 mL of PBS at RT for 6, 12, 24, 48, and 72 h prior to fixation. Following autolysis, pellets were centrifuged (300× *g*, 4 °C, 5 min), the supernatant was discarded, and the cells were resuspended in 1 mL of 10% NBF. The latter represents the most frequently used fixation concentration [8]. Fixation was carried out for a 6, 12, 24, 48, and 72 h at RT before paraffin embedding.

### 2.2. Light Microcopy

From each FFPE block, 2 µm-thick sections were prepared using a microtome. The first section of each block was stained with hematoxylin and eosin (HE) following a standardized protocol using an automated slide stainer (Tissue Stainer TST 44, Medite Medical GmbH, Burgdorf, Germany). HE-stained sections were evaluated semi-quantitatively after fixation in formalin at varying concentrations and durations. Nuclear and cytoplasmic morphology were assessed [12,64] with particular attention to staining and shrinkage artifacts. Tissue quality and suitability for subsequent diagnostic interpretation were scored as follows: 2 = good, 1 = moderate, 0 = poor. In the second experiment, HE-stained sections were analyzed to evaluate the effects of autolysis upon cell morphology. The microscopic evaluation of the HE-stained sections focused on nuclear alterations including swelling, loss of basophilia, karyorrhexis, karyolysis, and pyknosis, cytoplasmic changes such as vacuolization, swelling, and increased eosinophilia, as well as structural damage to the cell membrane, including cellular swelling and loss of membrane integrity [65,66]. To quantify the degree of autolytic degradation, a semi-quantitative decay score was applied. For each stained section, five high power fields (HPFs) were evaluated, and cells were categorized based on nuclear preservation into five grades: grade 1 representing normal nuclear morphology, grade 2 indicating pyknosis and mild karyorrhexis, grade 3 reflecting advanced karyorrhexis, grade 4 representing the absence of identifiable nuclei and grade 5 indicating complete loss of recognizable cellular structures. The decay score was calculated using the following formula: Decay score = (% cells with grade 1 × 1) + (% with grade 2 × 2) + (% with grade 3 × 3) + (% with grade 4 × 4) + (% with grade 5 × 5) [67,68].

### 2.3. Immunohistochemistry

CDV infection of DH82 CDV-Ond p.i. cells was confirmed by IHC using a monoclonal mouse antibody directed against CDV nucleoprotein [69]. Antibodies against IFN-β, Mx [46,69], ISG15, and pPKR [46,69] were used to detect activation of the type I IFN cascade. Data about used primary antibodies, dilutions, and pretreatments are shown in Table 1. IHC was performed according to de le Roi et al. (2025) [70]. Briefly, sections were deparaffinized twice in ROTI^®^Histol (Carl Roth, Cat.-No. 8028-48-6, Karlsruhe, Germany) and hydrogenated in a descending alcohol series. Subsequently, the endogenous peroxidase activity was blocked by incubating the sections for 30 min at RT in 85% ethanol with 0.5% H_2_O_2_ (Carl Roth, Cat.-No. 7722-84-1, Karlsruhe, Germany). The sections were washed twice for 5 min each in PBS at RT. Heat-induced antigen retrieval was performed. Therefore, sections were treated for 20 min in citrate buffer containing distilled water with 1 mol/L citric acid at 800 watts in a microwave. The sections were then washed again for 5 min at RT in PBS, followed by application of normal goat serum to the tissue to prevent non-specific binding. The primary antibody was then diluted with PBS and 1% albumin bovine fraction V (BSA, Serva Electrophoresis GmbH, Cat.-No. 9048-46-8, Heidelberg, Germany) in the indicated dilutions (Table 1) overnight at 4 °C. As negative controls, the primary antibodies were replaced by ascites fluid from non-immunized BALB/cJ mice and rabbit normal serum. The sera were diluted with PBS to the protein concentration of the used antibody. Positive controls included tissue sections from CDV-infected canine encephalitis cases for the CDV antibody, lung sections from influenza-infected mice exhibiting IFN-β-positive signals for the IFN-β antibody, and lung sections from SARS-CoV-2–infected hamsters as well as brain sections from CDV-infected dogs for antibodies targeting interferon-stimulated genes (Mx, ISG15, pPKR). After washing again twice in PBS for 5 min at RT, the secondary biotinylated goat-anti-rabbit or goat-anti-mouse antibody (diluted 1:200 in PBS) was applied to the sections for 45 min at RT. This was followed by application of the avidin-biotin complex (VECTASTAIN Elite ABC Kit, PK-6200, Vector Laboratories, Burlingame, CA, USA) according to the manufacturer’s protocol for 30 min at RT. Antigen–antibody binding was visualized by a five minute incubation in 3,3′-diaminobenzidine tetrahydrochloride (0.05%, Acros organics, AC112090050, Geel, Belgium) with addition of 0.03% hydrogen peroxide. Counterstaining was performed with acid hemalaun solution according to Mayer (Carl Roth, Cat.-No. T865.3, Karlsruhe, Germany). From the second experiment investigating the effects of autolysis and formalin fixation, only the sections that had been fixed in 10% NBF for 6 or 72 h after autolysis periods of 6, 12, 24, 48, and 72 h were stained.

The stained sections were digitized using the Olympus VS200 SlideView scanner (Olympus Life Science, Hamburg, Germany). IHC was analyzed using the open-source software QuPath (version 0.5.1) [71]. Within each cell pellet, a region of interest (ROI) measuring 1.5 mm^2^ was defined for subsequent image analysis. For each antibody, marker-specific threshold values were established and applied to quantify immunolabeled cells using automated positive cell detection within the defined ROI. The percentage of positive cells was calculated in relation to the total number of cells present in the ROI [72].

### 2.4. Statistics

Statistical analyses were performed using SAS^®^ statistical software, version 9.4M7, with SAS Studio Enterprise, version 3.8.2 (SAS Institute Inc., Cary, NC, USA). Graphs were created using GraphPad Prism, version 9.3.1 (GraphPad Software, San Diego, CA, USA) for Windows™. Each cell pellet was treated as an independent biological replicate, and three replicates were included for each experimental condition. A normal distribution was assessed using the Kolmogorov–Smirnov test, Cramér von Mises test and Anderson–Darling test. Variables that deviated from normality were square-root transformed, including ISG15 staining in infected cells under different formalin fixation conditions, IFN-β and pPKR staining in infected and non-infected cells following formalin fixation, CDV staining in infected cells exposed to autolysis and Mx, and IFN-β staining in infected and non-infected cells subjected to autolysis. For data that were normally distributed or reached a normal distribution after square root transformation, effects of factors were analyzed using a multifactorial ANOVA. Post-hoc pairwise multiple comparisons were performed using Least Squares Means with Tukey adjustment. Non-normally distributed data were converted to Wilcoxon scores, and significant differences were determined using the Kruskal–Wallis test. This included ISG15 staining in non-infected formalin-fixed cells, Mx and CDV nucleoprotein expression in infected and non-infected formalin-fixed cells, ISG15 expression under autolysis conditions, CDV staining in non-infected autolyzed cell pellets, pPKR expression in infected autolyzed cells, and decay scores across autolysis treatments. Pairwise two-sided multiple comparisons for non-parametric data were conducted using the Dwass–Steel–Critchlow–Fligner method. Statistical significance was accepted at *p*-values below 0.05.

## 3. Results

### 3.1. Histomorphological Assessment of DH82 Cells Under Different Fixation and Autolysis Conditions

In the experiment analyzing the morphology of DH82 cells under different fixation conditions, DH82 cells, regardless of CDV infection, appeared as large, round cells (20 to 50 µm) with moderate to abundant eosinophilic cytoplasm, often granular or vacuolated. Their nuclei were eccentrically located, large, bean-shaped (8–12 µm), occasionally multinucleated with finely stippled chromatin and one to three large, prominent eosinophilic nucleoli. Some cells showed phagocytosis. The mitotic index was approximately 25 mitotic figures per 10 HPFs at 40× magnification (2.37 mm^2^). No cytopathic effect was observed as a result of CDV infection. Light microscopic assessment of HE-stained sections confirmed, that fixation time and NBF concentration did not influence cell morphology. Cells consistently exhibited a prominent eosinophilic cytoplasm and intense nuclear basophilia with preserved morphology and without evidence of color fading or shrinkage artifacts (score 2). These observations raise the question of whether morphological alterations may become more pronounced when fixation is preceded by varying periods of autolysis.

**Figure 2:**
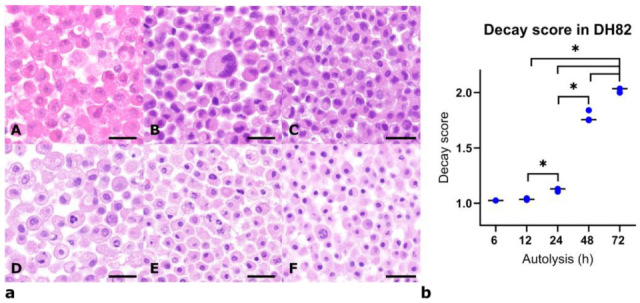
(**a**) Hematoxylin and eosin (HE)-stained sections of the canine histiocytic sarcoma cell line DH82 (non-infected controls) after 0 h (**A**), 6 h (**B**), 12 h (**C**), 24 h (**D**), 48 h (**E**), and 72 h (**F**) of autolysis, display progressive morphological alterations. Representative images demonstrate decreased cytoplasmic volume and nuclear pyknosis with prolonged autolysis characterized by pyknosis (**B**,**C**), karyorrhexis (**D**,**E**) and karyolysis (**E**). Scale bars: 50 µm. (**b**) Aligned dot plots of decay scores after 6, 12, 24, 48, and 72 h of autolysis. Each dot represents an individual measurement, and the horizontal line indicates the median. Statistical significance was determined by the Kruskal–Wallis test with post-hoc comparisons (* *p* < 0.05). *n* = 3 cell pellets per group.

In the experiment analyzing the combined effects of autolysis and fixation, progressive morphological alterations were observed with increasing duration of autolysis (Figure 2a(A–F)). Nuclear changes were characterized by an initial stage of karyorrhexis, followed by pyknosis and occasionally karyolysis. Cytoplasmic changes included increased eosinophilia, reduced granularity, dropped vacuolization and decreased volume. The Kruskal–Wallis test indicated that autolysis had a significant effect on the decay score in both non-infected and infected cells. All pairwise comparisons between autolysis times were significant, except for 6 h vs. 12 h and 48 h vs. 72 h in infected cells (Figure 2b). In addition, statistical analysis revealed a significant effect of both fixation time and infection status on the decay score (*p* < 0.05). In general, short fixation (6 h) after autolysis led to significantly higher decay scores compared to longer fixation (72 h), in both infected and non-infected cells. Moreover, infected cells showed greater degeneration than non-infected cells after prolonged autolysis combined with short fixation, while non-infected cells fixed for 72 h after 24 h autolysis displayed higher decay scores than infected cells under the same conditions. Overall, these data indicate that autolysis affects cell morphology regardless of the used formalin concentration. It remains to be clarified whether these pre-analytical variables might also influence the immunohistochemical detection of viral and host antigens.

### 3.2. Detection of CDV Nucleoprotein Under Variable Conditions

**Figure 3:**
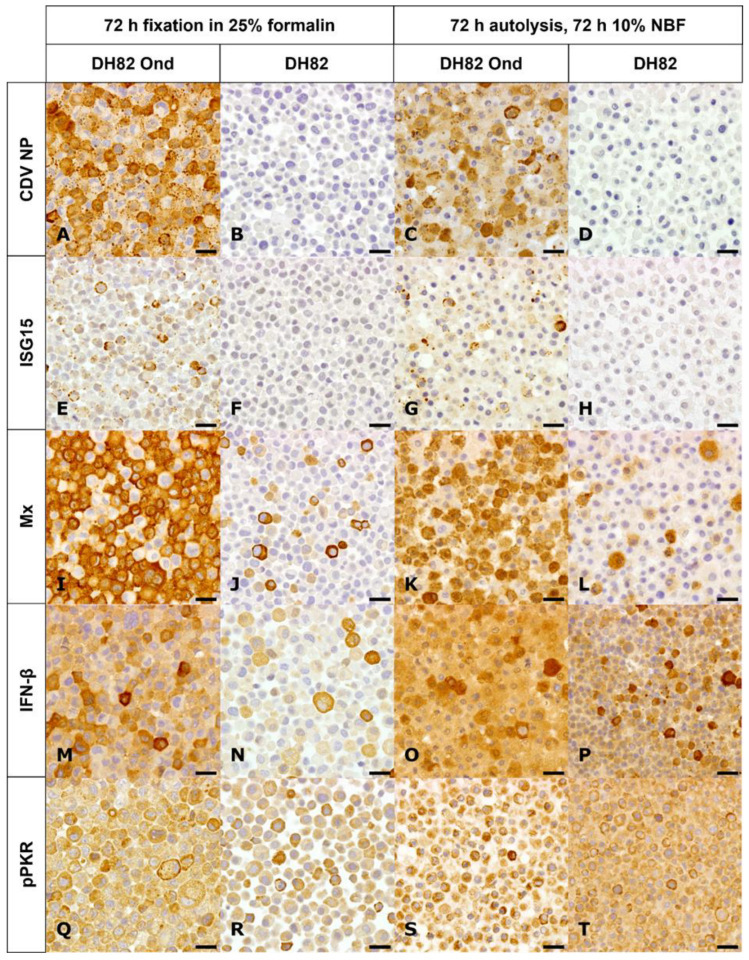
Immunohistochemical staining of persistently canine distemper virus (CDV; *Morbillivirus canis*) strain “Onderstepoort” (CDV-Ond)-infected DH82 cells (**A**,**C**,**E**,**G**,**I**,**K**,**M**,**O**,**Q**,**S**) and non-infected DH82 cells (**B**,**D**,**F**,**H**,**J**,**L**,**N**,**P**,**R**,**T**) under different pre-analytical conditions. (**A**,**B**,**E**,**F**,**I**,**J**,**M**,**N**,**Q**), and (**R**) show cells fixed in 25% neutral buffered formalin (NBF) for 72 h without prior autolysis, whereas panels (**C**,**D**,**G**,**H**,**K**,**L**,**O**,**P**,**S**), and (**T**) show cells subjected to 72 h autolysis before fixation in 10% NBF for 72 h. (**A**–**D**) CDV nucleoprotein (NP): Infected cells displayed a strong, diffuse to granular cytoplasmic staining of CDV-nucleoprotein antigen, occasionally with perinuclear accentuation, while non-infected cells were negative. Staining intensity was high across fixation conditions. (**E**–**H**) Interferon stimulated gene 15 (ISG15): Infected cells exhibited intense, cytoplasmic staining, whereas non-infected cells were negative. The signal was well preserved across formalin concentrations and autolysis. (**I**–**L**) Myxovirus resistance protein (Mx): Infected cells showed strong cytoplasmic staining, no influence of fixation parameters and slight attenuation after autolysis in non-infected cells. Non-infected cells exhibited less staining. (**M**–**P**) Interferon-β (IFN-β): Weak to moderate cytoplasmic staining was detected in infected cells, as well as in non-infected controls. (**Q**–**T**) Phosphorylated protein kinase R (pPKR): Variable cytoplasmic and perinuclear staining was observed in both infected and non-infected cells, with sporadically significant stronger signal in infected cells (after 12 and 48 h of autolysis and 6 h formalin fixation). Detection was inconsistent after autolysis and generally more robust in non-autolyzed pellets. Scale bar = 20 µm.

Immunohistochemical staining for the CDV nucleoprotein antigen revealed approximately 100% immunopositivity in persistently infected DH82 cells and 0% in non-infected controls (Figure 3A–D). In general, the immunohistochemical signal was localized in the cytoplasm of DH82 cells, without a background signal and exhibited a fine- to coarse-granular pattern (Figure 3A,B). Kruskal–Wallis test revealed no significant differences in the number of immuno-positive cells at the various formalin concentrations and after different fixation times (*p* > 0.05, Supplementary Figure S1).

**Figure 4:**
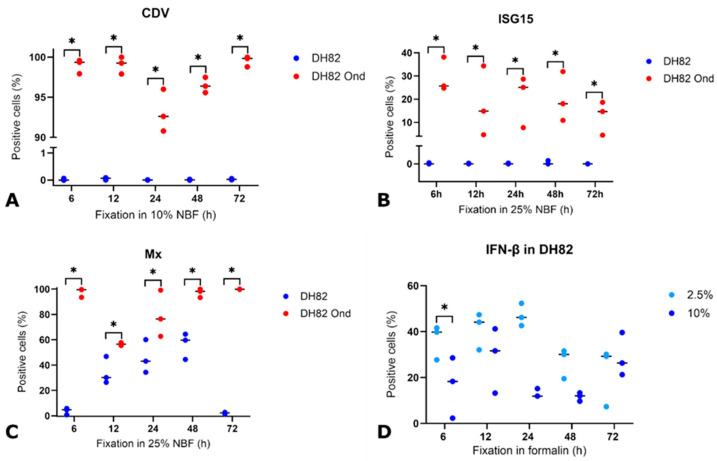
Immunohistochemical expression of (**A**) canine distemper virus (now *Morbillivirus canis*, CDV) nucleoprotein antigen, (**B**) interferon-stimulated gene 15 (ISG15), (**C**) myxovirus resistance protein (Mx), and (**D**) interferon-β (IFN-β) in the canine histiocytic sarcoma cell line DH82 after fixation for 6, 12, 24, 48 or 72 hours (h) in 10% (CDV), 25% (Mx, ISG15) or 2.5%, 10% and 25% (IFN-β) neutral buffered formalin (NBF). Data are presented as aligned dot plots showing the percentage of immuno-positive cells in non-infected and CDV-Onderstepoort (Ond)-infected cells. Each dot represents an individual measurement, and the horizontal line indicates the median. Statistical significance was determined by the Kruskal–Wallis test (Mx, CDV) or ANOVA (ISG15, IFN-β) with post-hoc comparisons using the Tukey adjustment (* *p* < 0.05). *n* = 3 cell pellets per group.

Generally, autolysis led to a slight reduction in signal intensity, a finer granular pattern and minimally increased background staining (Figure 3C,D). Statistical analysis revealed that autolysis had no effect on CDV expression (*p* > 0.05, Supplementary Figure S5). Moreover, across all conditions, CDV-infected cells showed significantly more CDV-positive cells than non-infected controls (*p* < 0.05, Figure 3C,D and Figure 4A). These observations suggest that CDV detection is relatively insensitive to variations in fixation time or formalin concentration and autolysis within this experimental system. These findings raise the question whether a similarly stable and reliable detection can be achieved for the selected innate immune response antigens.

### 3.3. Detection and Expression of ISG15, Mx, IFN-β, Phosphorylated PKR (pPKR), and Its Stability Under Various Pre-Fixation Conditions

**Figure 5:**
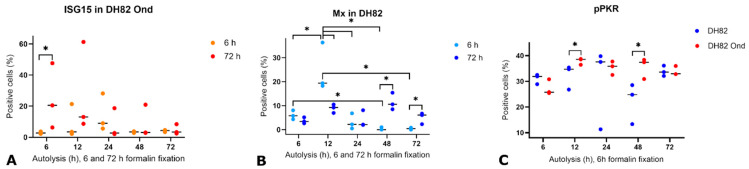
Immunohistochemical expression of (**A**) interferon-stimulated gene 15 (ISG15), (**B**) myxovirus resistance protein (Mx), and (**C**) phosphorylated protein kinase R (pPKR) in the canine histiocytic sarcoma cell line DH82 after autolysis for 6, 12, 24, 48, or 72 hours (h) and fixation for 6 or 72 h in 10% neutral buffered formalin. Data are presented as aligned dot plots showing the percentage of immunopositive cells in non-infected and persistently canine distemper virus (CDV; *Morbillivirus canis*) strain “Onderstepoort” (Ond) infected cells. Each dot represents an individual measurement, and the horizontal line indicates the median. Statistical significance was determined by the Kruskal–Wallis test (ISG15, pPKR) and ANOVA (Mx) with post-hoc comparisons using the Tukey adjustment (* *p* < 0.05). *n* = 3 cell pellets per group.

ISG15 expression was observed in 19% of CDV-infected DH82 cells and 0% of controls. Overall, the signal was cytoplasmic, granular, and of moderate intensity, with minimal background staining (Figure 3E,F). Statistical analysis revealed no systematic signal loss due to different formalin concentrations or fixation times (*p* > 0.05, Supplementary Figure S2). Autolysis had an impact on ISG15 detection and resulted in general in increased background staining and a finer granular distribution (Figure 3G,H). Statistical analysis confirmed that infected cells autolyzed for 6 h and fixed for 72 h had significantly higher ISG15 immunopositivity than those fixed for only 6 h (*p* < 0.05, Figure 5A). Infected cells demonstrated significantly more ISG15-positive cells than non-infected cells across all combinations of fixation (*p* < 0.05, Figure 4B) and autolysis, with autolysis slightly affecting the staining pattern and intensity. It was therefore of interest to investigate whether this stability also applied to other ISGs such as Mx.

Mx expression averaged 89% in CDV-infected DH82 cells and 16% in non-infected cells. Overall, the cytoplasmic signal ranged from granular to homogeneous and lacked background staining (Figure 3I,J). No significant differences of immunopositivity were associated with the fixation time or formalin concentration (*p* > 0.05, Supplementary Figure S3A,B). Generally, autolysis led to increasingly granular staining and a slightly elevated background signal (Figure 3K,L). A significant three-way interaction among infection, autolysis duration, and fixation time was observed (*p* < 0.05). In non-infected cells, 12 h autolysis with 6 h fixation yielded significantly higher numbers of Mx-positive cells than 6, 24, and 48 h autolysis with the same fixation (Figure 5B). Similarly, the number of Mx-positive non-infected cells was higher after 12 h autolysis with 6 h fixation than after 72 h fixation (Figure 5B). Longer fixation (72 h) after 48 or 72 h autolysis significantly increased Mx immunopositivity compared to shorter fixation (6 h) after the same autolysis periods. Conversely, prolonged autolysis (48 and 72 h) combined with only 6 h fixation resulted in significantly lower Mx-positive cell counts compared to shorter autolysis (Figure 5B). Overall, infected cells demonstrated significantly more Mx-positive cells than non-infected DH82 cells (Figure 4C), independent of autolysis, fixation time or formalin concentration. Autolysis affected the staining intensity of Mx, and the longer fixation time preserved immunopositivity in non-infected cells. These observations raise the question of whether upstream mediators of interferon responses, such as IFN-β, would show a similar reaction to pre-analytical variation.

IFN-β expression was found in approximately 24% of CDV-infected and non-infected DH82 cells. The cytoplasmic signal was predominantly granular to homogeneous with moderate to strong background staining (Figure 3M,N). Statistical analysis of IFN-β-stained sections revealed a significant two-way interaction between infection status and formalin concentration (*p* < 0.05). Non-infected cells fixed for 6 h in 2.5% formalin exhibited higher numbers of immuno-positive cells compared to cells fixed in 10% formalin (Figure 4D). Autolysis was largely associated with a mild decreased staining intensity (Figure 3O,P). Statistical analysis revealed that autolysis had no significant effect on IFN-β expression (*p* > 0.05, Supplementary Figure S6A,B). Finally, it remained to be determined whether pPKR is equally affected by fixation and autolysis.

Although pPKR expression was detected in 37% of infected and 41% of non-infected DH82 cells, significant differences between the two groups were observed depending on the treatment conditions. In general, the signal was weak to moderate and showed a granular to homogeneous cytoplasmic pattern with moderate to strong background staining (Figure 3Q,R). A significant three-way interaction among infection, fixation time, and formalin concentration was observed (*p* < 0.05). Specifically, infected cells fixed for 48 h in 2.5% formalin showed significantly higher immunopositivity than cells fixed for 72 h in 2.5% (Supplementary Figure S4). pPKR immunostaining after autolysis and formalin fixation generally showed increased background staining (Figure 3S,T). Statistical analysis revealed that infection significantly influenced immunopositivity (*p* < 0.05), with infected cells showing higher numbers of positive cells than non-infected controls after 12 or 48 h of autolysis and 6 h of fixation (Figure 5C). Overall, pPKR was detected in both CDV-infected and non-infected DH82 cells, with infection increasing immunopositivity under specific autolysis and fixation conditions, while autolysis slightly reduced staining.

## 4. Discussion

The present study investigated the effects of autolysis, the formalin concentration, and fixation time on histomorphology and immunohistochemical detection of the CDV antigen and components of the innate immune response in DH82 cells. The aim of this study was to mimic conditions encountered during necropsy, providing reliable data for future retrospective analysis. Overall, autolysis has a greater impact on cell morphology than on the reliable immunohistochemical detection of viral and host antigens whereas formalin concentration and fixation duration have minimal impact on antigenicity. Moreover, CDV antigen detection proved stable across all conditions, highlighting the diagnostic reliability. The consistent ISG15 and Mx upregulation and detection further supports their potential usefulness as virus-sensing markers, even under suboptimal pre-analytical conditions.

Analysis of the formalin concentration and fixation duration revealed no significant impact on HE staining. Likewise, the immunohistochemical detection of the CDV nucleo-protein, ISG15, and Mx antigen was not affected. However, for IFN-β and pPKR staining, a significant higher number of immunopositive cells was observed at lower formalin concentrations and shorter fixation times. Formalin fixation can alter the protein structure through its cross-linking effects. This may damage epitopes or restrict their accessibility, which may reduce immunoreactivity [6,11]. In line with this, stronger immunoreactivity for IFN-β and pPKR was observed at lower formalin concentrations and shorter fixation times in the present study. These findings are consistent with a previous study that evaluated the effects of five different formalin concentrations (ranging from 0.25% to 4%) on IHC for glutaminase and nociceptive neuron markers. The authors suggested that a 0.5% formalin concentration represents the optimal condition [73]. Conversely, another study reported no significant effect of low formalin concentrations (1–5%) for 24 and 48 h on HE staining or IHC [64]. However, inadequate fixation whether due to shortened duration or reduced formalin concentration also poses risks [6]. Formalin penetrates tissue slowly (1 cm^3^ in 24 h), potentially causing autolysis in central tissue regions while peripheral tissue areas are fixed [12]. Subsequent alcohol fixation during paraffin embedding may then lead to tissue shrinkage [13]. Comparable alterations were not observed in the present study, even with short fixation times (6 h) in 2.5% formalin, likely due to the small size of the cell pellet (0.5 cm^3^), which allowed rapid and uniform formalin penetration. Some studies have suggested that under-fixation may be more problematic than over-fixation. They suggest 8 days of fixation in 10% NBF for good immunohistochemical results, as under-fixation can lead to increased background staining [20,74]. Though under-fixation can lead to tissue shrinkage, increased background staining or central autolysis, prolonged fixation or fixation using highly concentrated formalin can cause increased cross-linking. This may result in an additional loss or masking of epitopes [6,9,11]. Appropriate antigen retrieval can partially reverse these effects [21]. Notably, in the present study, no complete loss in immunoreactivity was observed for IFN-β or pPKR, even after prolonged fixation for 72 h in 25% formalin, suggesting resistance of the respective epitopes or the antibodies under the tested conditions. It remains possible, that longer fixation could lead to signal loss. This highlights that the susceptibility to formalin is likely marker dependent. These findings are particularly relevant for retrospective analyses of archived material. They indicate that reliable detection of these innate immune markers can be achieved even under suboptimal pre-analytical conditions. Previous investigations of prolonged fixation (up to 12 weeks) reported moderate to strong immunoreactivity for over 80 antibodies [6,9]. In contrast, a study on extended fixation (up to 15 days) for immunohistochemical detection of porcine reproductive and respiratory syndrome (PRRS) virus observed decreased immunoreactivity already at day 3 [75], supporting the notion that formalin sensitivity is epitope and/or antibody dependent [6]. Furthermore, it should be considered that, in addition to formalin concentration and fixation time, other factors can influence the outcomes of histological and immunohistochemical analyses, including the volume of formalin used and the temperature during fixation [8,12]. However, these two parameters were not investigated within the present study. In addition, differences between conventional formalin fixation and modified fixation protocols as well as the use of alternative fixatives were not examined in the present study. Previous investigations have suggested that storing formalin-fixed tissues in ethanol may better preserve immunoreactivity than prolonged storage in formalin [76]. Moreover, fixatives such as glyoxal acid-free (GAF) [77] or Amber fixative [78] have been reported to produce histological and immunohistochemical results comparable to standard formalin fixation.

In the present study the combined effects of autolysis and formalin fixation were analyzed. Autolysis was associated with progressive morphological alterations, including nuclear changes such as karyorrhexis, karyolysis and pyknosis and cytoplasmic alterations such as vacuolization, swelling and increased eosinophilia as described by others [66,79]. Autolysis leads to the disintegration of cellular components, making it difficult to distinguish tissue architecture and specific cell types [24,80]. The degree of autolysis varies between tissue and cell types, dependent on the amount of hydrolytic enzymes, lysosomes and peroxisomes [22,28]. In the present study, objective assessment of autolytic changes using the decay score on HE stained sections revealed alterations up to grade 3 (advanced karyorrhexis) of 5 (no cell visible) after 72 h of autolysis. In this *in vitro* model, cell pellets were left at room temperature for periods ranging from 6–72 h. This approach allows the analysis of autolytic changes without external influences. However, it should be noted that this model only partially reflects postmortem changes occurring in tissues. Using a cell line cannot capture the effects of stromal components or vascular supply. Moreover, the model did not account for variations in temperature, humidity, and pH during auto-lysis, differences in tissue thickness, or the impact of bacterial activity on tissue degradation. Similarly, a previous study has suggested that cell lines are less sensitive to autolysis, as they are not part of a tissue containing inflammatory cells and stroma that produce and release degrading enzymes [81]. In contrast, another study demonstrated rapid autolysis in the pancreas, with high decay scores within 5 h postmortem [67]. At the protein level, autolysis results in both specific and nonspecific proteolysis and dephosphorylation [82]. This compromises immunohistochemical analyses, as autolysis alters the tertiary structure of proteins, often resulting in a loss of immunoreactivity due to protein denaturation [23,28]. In the present study, autolysis manifested in some cases as reduced signal intensity, increased background staining, and partially altered staining patterns, while overall immunoreactivity was often preserved. Similar findings were reported in a study investigating the effects of autolysis (up to 72 h) on IHC for the characterization of central nervous system cells [65]. In contrast, in the present study, higher immunopositivity for Mx and ISG15 was detected after short autolysis periods, with loss of staining intensity upon longer autolysis. These findings are consistent with studies demonstrating decreased immunoreactivity for multiple antigens with prolonged autolysis [22,28,65,68,83]. This indicates that autolysis results in progressive protein degradation and epitope loss [28]. The extent of this process appears to be tissue and cell specific and can affect all protein classes and antigens [22,83]. However, phosphorylated epitopes are particularly susceptible to degradation and delayed phosphatase inactivation and tend to lose their immunoreactivity rapidly [12,26]. Surprisingly, in the present study, pPKR expression did not vary with autolysis, suggesting differential stability of this phosphorylated protein compared to others.

The 100% infection rate observed in this study underscores the high permissiveness of DH82 cells to CDV strain Onderstepoort (Ond), consistent with previous reports [63]. The significantly increased immunopositivity of ISG15, Mx, and, to a lesser extent, pPKR in infected cells strongly indicates the activation of an antiviral response, most likely mediated through the interferon signaling pathway [84]. During viral infection, viral replication intermediates, particularly dsRNA are detected by PRRs of the innate immune system, including PKR [85]. This recognition initiates a signaling cascade that typically culminates in the expression of IFN-β [37,86]. IFN-β then acts autocrine and paracrine by binding to IFN receptors, which activates the Janus kinase 1-signal transducer and activator of transcription (JAK-STAT) pathway. This activation induces the transcription of ISGs [39,86,87]. Notably, Mx is considered a reliable surrogate marker of IFN-β activity, as its expression is almost exclusively induced by type I IFNs [88]. In the present study, CDV infection led to significantly enhanced expression of Mx and ISG15 in DH82 cells. These findings are consistent with earlier reports of robust Mx upregulation in CDV-infected alveolar macrophages and in cases of canine distemper virus encephalitis [69,89]. Similarly, RNA sequencing analyses of CDV-infected DH82 cells revealed significant upregulation of various ISGs, including Mx and ISG15 [61]. In human medicine, ISG expression patterns are increasingly used as biomarkers to differentiate between viral and bacterial infections in febrile patients [49,88,90,91,92]. The pronounced upregulation and stable expression of Mx and ISG15 across diverse pre-analytical conditions highlight their robustness and suitability as potential virus-sensing tools.

In the present study, increased pPKR expression in infected compared to non-infected cells was observed only sporadically, which was unexpected. A more pronounced induction of pPKR would have been anticipated in CDV-Ond-infected cells. Several potential mechanisms could explain these patterns, but these were not assessed in the present study and therefore remain speculative. Previous studies have shown that attenuated CDV strains have defective C proteins, which lead to elevated levels of dsRNA, thereby strongly activating PKR [86]. Whether this mechanism also applies to the Ond strain, remains unclear. On the other hand, expression of C and V proteins encoded by the CDV genome is known to suppress PKR activation, which may account for the limited pPKR response observed in the present study [84]. The absence of a clear difference in pPKR between infected and non-infected cells may be also explained by the upregulation of PKR during mitotic activity, particularly in the M phase of the cell cycle [85,93]. Since DH82 cells are rapidly dividing irrespective of their infection status, high levels of pPKR may obscure infection-related differences. Secondly, PKR is constitutively expressed at basal levels in all tissues and is further induced by type I and type III IFNs [47]. Since only IFN-β expression was measured, potential PKR induction by IFN-α or IFN-III cannot be excluded. Moreover, in the present study, only the levels of phosphorylated PKR were determined, whereas the total PKR was not assessed.

In line with this, in the present study, IFN-β expression was found to be similarly high in both infected and non-infected cells, despite a marked increase in ISG expression among infected cells. Multiple possible explanations may account for these observations, although they are currently hypothetical. One possibility is that although IFN-β was present at detectable levels, its concentration was insufficient to exceed the autocrine activation threshold required for robust signaling. Alternatively, DH82 cells being of tumor origin may constitutively exhibit elevated basal IFN expression, a phenomenon previously reported in other tumors [94,95]. Furthermore, cellular stress during culture hand-ling, including oxidative or mechanical stress, has been shown to trigger IFN expression and could also play a role here [96]. In CDV-Ond-infected DH82 cells, expression of the C and V proteins of the CDV genome interfere with the activation of the type I IFN signaling pathway [84]. Previous studies have demonstrated that these proteins are capable of blocking specific PRRs, including PKR [37,84,97]. They can disrupt downstream signaling components following viral recognition [84]. Additionally, they prevent the activation and nuclear translocation of IFN regulatory factors (IRF) 3 and 7 [84]. These proteins also inhibit the JAK-STAT signaling cascade [84,98]. It should also be considered that ISG activation can be induced by other type I interferons, such as IFN-α [39]. The expression of IFN-α was not assessed in the present study. Finally, it cannot be excluded that the observed upregulation of ISG15 and Mx in infected DH82 cells occurred via IFN-independent mechanisms. Several pathways, including direct activation through IRF3 or NF-κB, can drive ISG expression without the involvement of secreted interferon [39,61]. It is therefore possible that ISG induction is not directly associated with detected IFN-β expression. Overall, the mechanisms underlying the limited pPKR and IFN-β responses observed here remain hypothetical and need further investigation.

A major strength of this work lies in the controlled evaluation of multiple antigens under conditions that closely resemble those encountered in routine necropsy settings, all within a single cell line. This standardized approach minimizes variability introduced by tissue heterogeneity and enables a focused analysis of pre-analytical factors. The results of the present study allow some conclusions to be drawn for practical diagnostics. Notably, markers such as CDV, ISG15, and Mx remained detectable even after 72 h of autolysis, suggesting that modest delays in fixation of small samples may not completely prevent immunohistochemical detection. Furthermore, variations in formalin fixation parameters appeared to have only a minimal impact on histological and immunohistochemical outcomes. It should be emphasized, however, that these findings are based on the analysis of a single cell line under highly controlled, artificial conditions. Several other limitations should be acknowledged. First, the use of a single *in vitro* cell model inherently restricts the generalizability of the findings, as immortalized cells may not fully reflect the biology of primary host cells or tissues. Although DH82 cells represent a valuable macrophage *in vitro* model for investigating early innate immune response signaling, they cannot recapitulate the complexity of the systemic antiviral response in tissues and organs or the organism. Consequently, the findings of this study should be interpreted as insights into cellular mechanisms rather than as a complete depiction of the host immune response. In addition, results obtained from the immunohistochemical staining of DH82 cells may not fully reflect the staining behavior in tissue sections. The present study did not include experimental validation of fixation parameters or immunostaining characteristics in animal tissue samples, which reflects a crucial limitation that needs to be considered before translating the present findings to *in vivo* reaction patterns. Moreover, the results were obtained using IHC alone, and further confirmation with quantitative methods such as quantitative polymerase chain reaction (PCR) or Western blot would strengthen the reliability of the findings. Future work will be necessary to validate these observations and to determine their relevance at the tissue and organism levels. Furthermore, DH82 cells are known to exhibit substantial variability in morphology and expression profiles depending on the passage number used [60]. To minimize this effect, closely related cell passages were used. In addition, as a tumor-derived cell line, DH82 cells likely possess a higher mutation rate than primary cells, which could affect expression levels [60]. Therefore, the expression of markers should be interpreted with caution. Future studies are needed to validate the potential utility of Mx and ISG15 as virus-sensing tools in tissue samples from different animal species affected by viral and non-viral inflammatory diseases. Such investigations would help to assess whether the robustness of Mx and ISG15 detection found in this study translates to physiologically relevant conditions.

## 5. Conclusions

Autolysis substantially affected histomorphology and, to a moderate extent, immunostaining characteristics, whereas variation in the formalin concentration and fixation time had comparatively minor effects. Detection of the CDV antigen was generally maintained even under suboptimal pre-analytical conditions, suggesting high robustness. Moreover, Mx and ISG15 were consistently upregulated in infected DH82 cells across different fixation and autolysis scenarios, which highlights their potential as virus-sensing markers. These observations are derived from a single cell line and should therefore be interpreted and extrapolated with caution. Results obtained from DH82 cells may not fully reflect staining behavior in tissue sections. The extent to which these findings apply to tissue samples or real necropsy conditions requires further validation. Taken together, these findings provide initial guidance for sample handling protocols to ensure reliable detection of viral antigens and immune markers in both diagnostic and research settings.

## Figures and Tables

**Table 1:** Data on used primary antibodies, applied dilutions, secondary antibodies, and applied demasking and pretreatment methods.

	Demasking	Blocking Serum	Primary Antibody, Source	Secondary Antibody	ABC	DAB
CDV nucleoprotein	20′ citrate buffer, microwave (800 W), 20′ cooling at RT	120 µL goat normal serum, Dilution: 1:5	D110, Prof. Dr. Zurbriggen, University of Bern, Switzerland, mouse monoclonal Dilution: 1:800	Goat-anti-Mouse IgG (H+L), biotinylated, VectorLab, Newark, CA, USA	120 µL/ slide; 1000 µL PBS + 15 µL reagent A + 15 µL reagent B	0.1 g DAB, 200 mL PBS, 250 µL H_2_O_2_
Interferon-β	20′ citrate buffer, microwave (800 W), 20′ cooling at RT		IFN beta antibody (PA5-102429), Invitrogen, Waltham, MA, USA, Lot #ZC4272091, rabbit polyclonal Dilution: 1:5000	Goat-anti-Rabbit IgG (H+L), biotinylated, VectorLab, Newark, CA, USA		
Myxovirus resistance protein	20′ citrate buffer, microwave (800 W), 20′ cooling at RT		M143, Prof. Dr. Haller and PD Dr. Koch, University Medical Center Freiburg, Germany, mouse monoclonal Dilution: 1:500	Goat-anti-Mouse IgG (H+L), biotinylated, VectorLab, Newark, CA, USA		
Phosphorylated protein kinase R	20′ citrate buffer, microwave (800 W), 20′ cooling at RT		pPKR, phosphor T446, Abcam Inc., Cambridge, MA, USA, Ab32036, rabbit monoclonal Dilution: 1:8000	Goat-anti-Rabbit IgG (H+L), biotinylated, VectorLab, Newark, CA, USA		
Interferon stimulated gene 15	20′ citrate buffer, microwave (800 W), 20′ cooling at RT		ISG15 (F-9), Santa Cruz Biotechnology, Santa Cruz, CA, USA, Sc-50366, mouse monoclonal Dilution: 1:1600	Goat-anti-Mouse IgG (H+L), biotinylated, VectorLab, Newark, CA, USA		

20′ = 20 min; ABC = avidin-biotin complex; DAB = 3,3′-diaminobenzidine tetrahydrochloride; H+L = heavy and light chains; IgG = immunoglobulin G; PBS = phosphate buffered saline; RT = room temperature; W = watt.

## Data Availability

The original contributions presented in this study are included in the article and its Supplementary Material. Further inquiries can be directed to the corresponding author.

## Supplementary Materials

The following supporting information can be downloaded at: https://www.mdpi.com/article/10.3390/v17121575/s1, Figures S1–S6. Immunohistochemical analysis of CDV NP, ISG15, Mx, pPKR, and IFN-β in DH82 cells under different fixation times, formalin concentrations, and autolysis conditions. Data as aligned dot plots, median and statistical analyses as indicated. Supplementary Datasets S1 and S2. Summary tables of % immunopositive cells and decay scores for all markers under different fixation and autolysis conditions.
