# Supplementary material for: An In Vitro Approach to Evaluate the Impact of Autolysis and Formalin Fixation on the Detection of Canine Distemper Virus and Innate Immune Response Antigens
Source: Viruses. 2025 Dec 2;17(12):1575. doi: 10.3390/v17121575 (PMC12737447; doi:10.3390/v17121575)
Supplement: Supplementary file 1 [file viruses-17-01575-s001.zip › Supplementary material_Figures.pdf]

Article

An *in vitro* approach to evaluate the impact of autolysis and formalin fixation on the detection of canine distemper virus and innate immune response antigens

Hannah Gerhards[1], Karl Rohn[2], Christina Puff[1], Wolfgang Baumgärtner[1*]

[1] Department of Pathology, University of Veterinary Medicine Foundation, Hannover, Germany; Hannah.gerhards@tiho-hannover.de; christina.puff@tiho-hannover.de
[2] Department of Biometry, Epidemiology and Data Management, University of Veterinary Medicine Foundation, Hannover, Germany; karl.rohn@tiho-hannover.de
* Correspondence: wolfgang.baumgaertner@tiho-hannover.de

Supplementary material: Figure S1 – S6

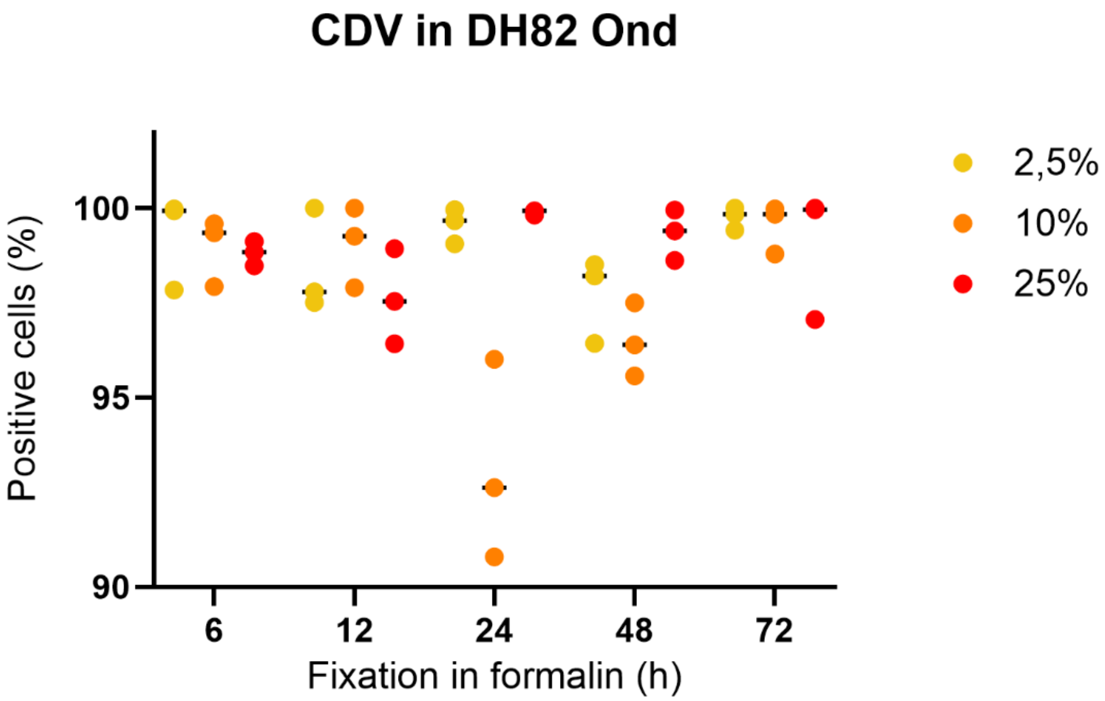

Figure S1. Immunohistochemical expression of canine distemper virus (now *Morbillivirus canis*, CDV) nucleoprotein in the canine histiocytic sarcoma cell line DH82 persistently infected with the strain Onderstepoort (Ond) after fixation for 6, 12, 24, 48 or 72 hours (h) in 2.5%, 10% and 25% neutral buffered formalin (NBF). Variation in formalin concentration did not significantly alter the immunohistochemical expression of CDV nucleoprotein. Data is presented as aligned dot plots showing the percentage of immunopositive cells in CDV-Onderstepoort (Ond) infected cells. Each dot represents an individual measurement and the horizontal line indicates the median. Statistical significance was determined by Kruskal–Wallis test with post-hoc comparisons using the Dwass-Steel-Critchlow-Fligner method ($p < 0.05$). $n = 3$ cell pellets per group.

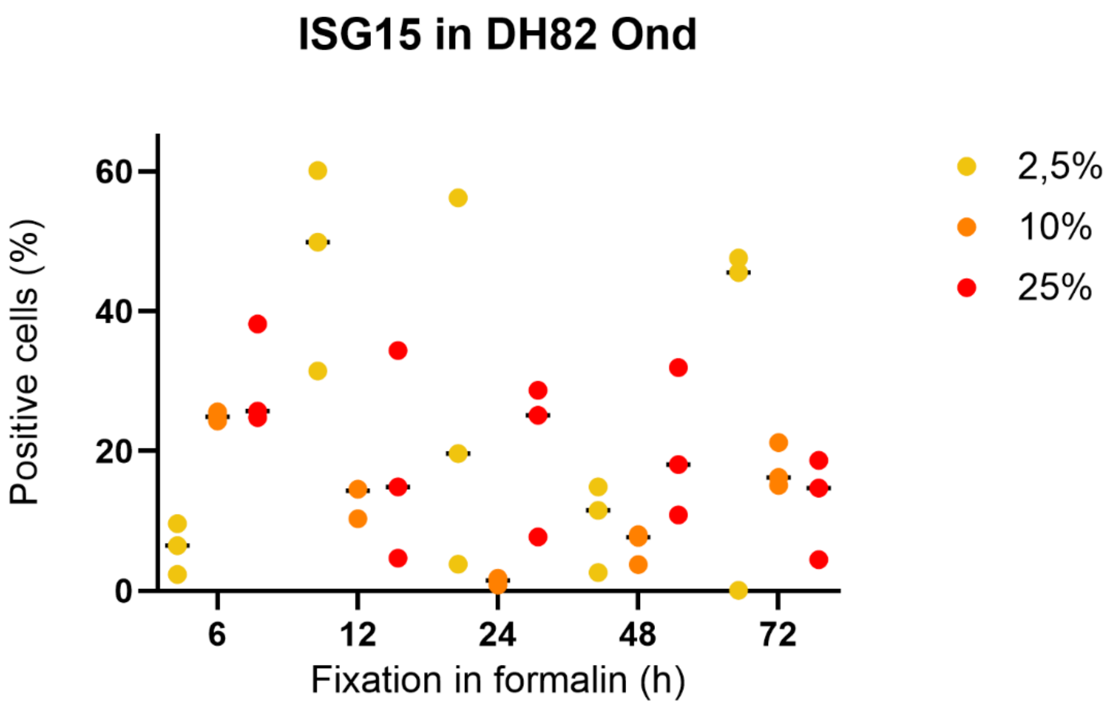

Figure S2. Immunohistochemical expression of interferon stimulated gene 15 (ISG15) in the canine histiocytic sarcoma cell line DH82 persistently infected with the canine distemper virus (now *Morbillivirus canis*, CDV) strain Onderstepoort (Ond) after fixation for 6, 12, 24, 48 or 72 hours (h) in 2.5%, 10% and 25% neutral buffered formalin (NBF). Variation in formalin concentration did not significantly alter the immunohistochemical expression of ISG15. Data is presented as aligned dot plots showing the percentage of immunopositive cells in CDV-Onderstepoort (Ond) infected cells. Each dot represents an individual measurement and the horizontal line indicates the median. Statistical significance was determined by ANOVA with post-hoc comparisons using Tukey adjustment (*p < 0.05). *n* = 3 cell pellets per group.

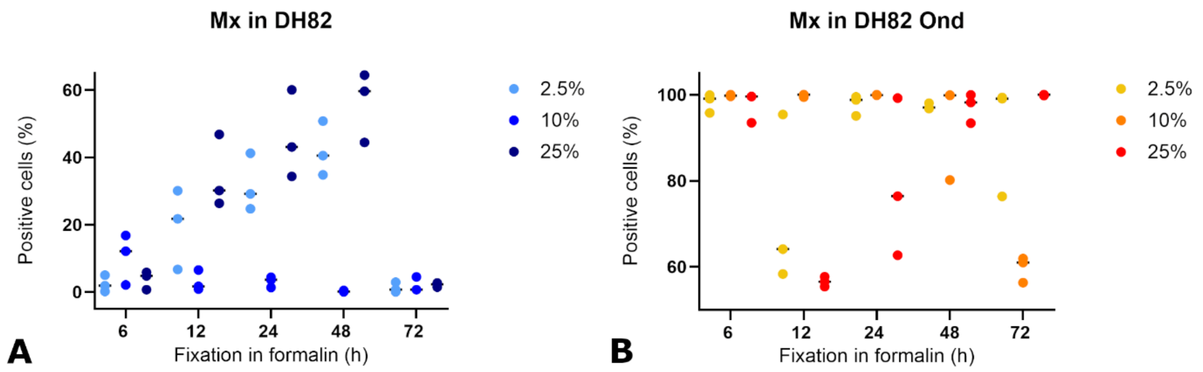

Figure S3. Immunohistochemical expression of myxovirus resistance protein (Mx) in the canine histiocytic sarcoma cell line DH82 after fixation for 6, 12, 24, 48 or 72 hours (h) in 2.5%, 10% and 25% neutral buffered formalin (NBF). Variation in formalin concentration did not significantly alter the immunohistochemical expression of Mx. Data is presented as aligned dot plots showing the percentage of immunopositive cells in **(A)** non-infected controls (DH82) and **(B)** Canine distemper virus (now *Morbillivirus canis*, CDV) Onderstepoort-infected cells (Ond). Each dot represents an individual measurement and the horizontal line

indicates the median. Statistical significance was determined by Kruskal–Wallis test with post-hoc comparisons using the Dwass-Steel-Critchlow-Fligner method ($p < 0.05$). $n = 3$ cell pellets per group.

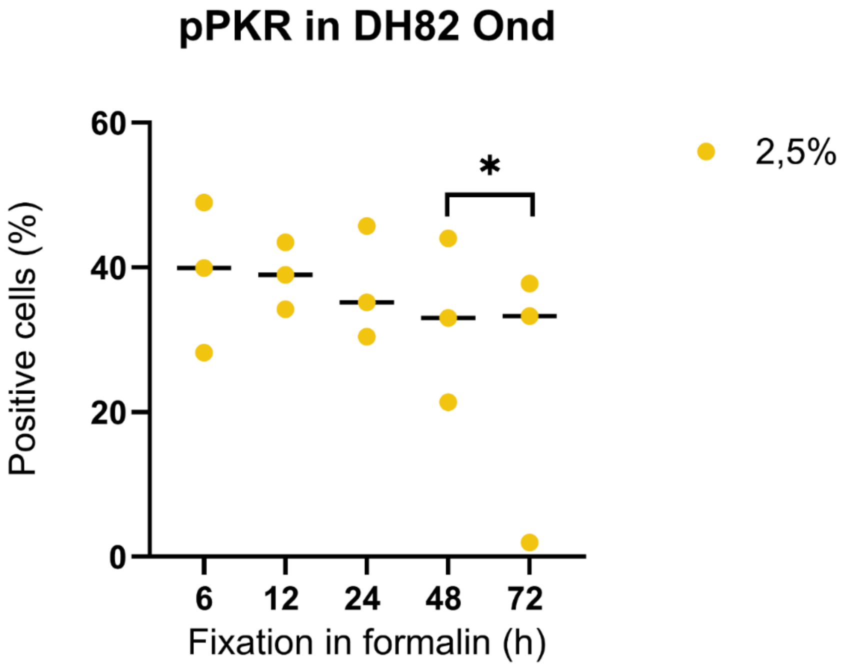

Figure S4. Immunohistochemical expression of phosphorylated protein kinase R (pPKR) in the canine histiocytic sarcoma cell line DH82 persistently with the canine distemper virus (now *Morbillivirus canis*, CDV) strain Onderstepoort (Ond) infected after fixation for 6, 12, 24, 48 or 72 hours (h) in 2.5% neutral buffered formalin (NBF). Data is presented as aligned dot plots showing the percentage of immunopositive cells. Infected cells fixed for 48 h in 2.5% formalin showed significantly higher immunopositivity than cells fixed for 72 h in 2.5%. Each dot represents an individual measurement and the horizontal line indicates the median. Statistical significance was determined by ANOVA with post-hoc comparisons using Tukey adjustment (*$p < 0.05$). $n = 3$ cell pellets per group.

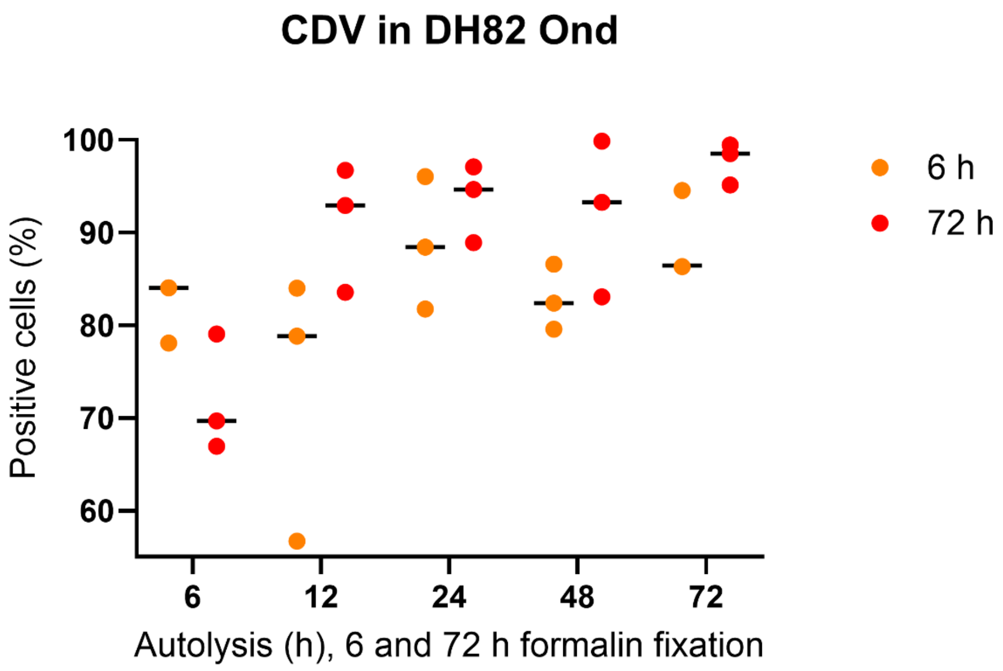

Figure S5. Immunohistochemical expression of canine distemper virus (now *Morbillivirus canis*, CDV) nucleoprotein in persistently with the strain Onderstepoort (Ond) infected canine histiocytic sarcoma cell line DH82 after autolysis for 6, 12, 24, 48 or 72 hours (h) and subsequent fixation in 10% neutral buffered formalin (NBF) for 6 h and 72 h. Data is presented as aligned dot plots showing the percentage of immunopositive cells. Variation in autolysis did not significantly alter the immunohistochemical expression of CDV. Each dot represents an individual measurement and the horizontal line indicates the median. Statistical significance was determined by ANOVA with post-hoc comparisons using Tukey adjustment (*$p < 0.05$). $n = 3$ cell pellets per group.

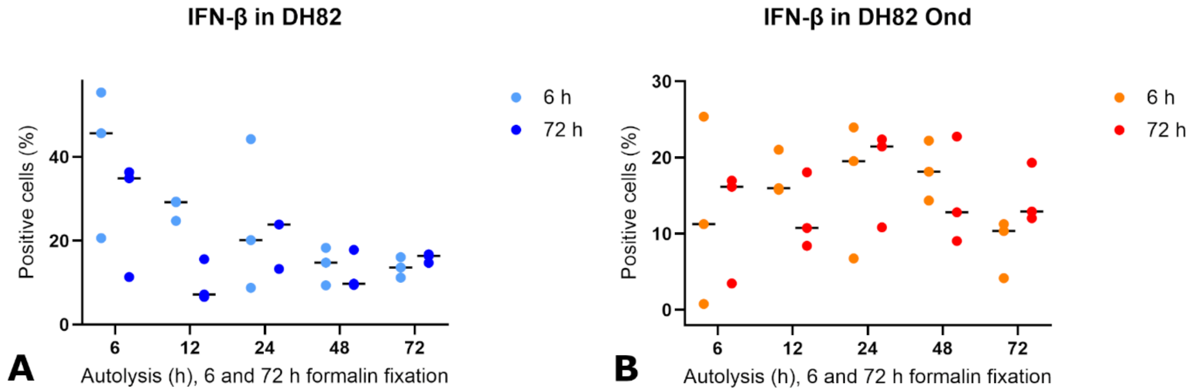

Figure S6. Immunohistochemical expression of interferon-β (IFN-β) in the canine histiocytic sarcoma cell line DH82 non-infected (**A**) and persistently with the canine distemper virus (now *Morbillivirus canis*, CDV) strain Onderstepoort (Ond) infected (**B**) after autolysis for 6, 12, 24, 48 or 72 hours (h) and subsequent fixation in 10% neutral buffered formalin (NBF) for 6 h and 72 h. Data is presented as aligned dot plots showing the percentage of immunopositive cells. Variation in autolysis did not significantly alter the immunohistochemical expression of IFN-β. Each dot represents an individual measurement and the horizontal line

indicates the median. Statistical significance was determined by Kruskal–Wallis test with post-hoc comparisons using the Dwass-Steel-Critchlow-Fligner method (*$p < 0.05$). $n = 3$ cell pellets per group.
