# Supplementary material for: An In Vitro Approach to Evaluate the Impact of Autolysis and Formalin Fixation on the Detection of Canine Distemper Virus and Innate Immune Response Antigens
Source: Viruses. 2025 Dec 2;17(12):1575. doi: 10.3390/v17121575 (PMC12737447; doi:10.3390/v17121575)
Supplement: Supplementary file 1 [file viruses-17-01575-s001.zip › Description of Supplementary Datasets.pdf]

Supplementary Dataset 1: A table summarizing the percentage of immunopositive cells after fixation in formalin across different durations (6 – 72 h) and concentrations (2.5 – 25%). The table displays infection status (1 = not infected; 2 = persistently infected with canine distemper virus (CDV; *Morbillivirus canis*), strain Onderstepoort (Ond)), formalin concentration, fixation time and the number of immunopositive cells for the markers canine distemper virus nucleoprotein (CDV NP), interferon-stimulated gene 15 (ISG15), myxovirus resistance protein (Mx), interferon-β (IFNβ), and phosphorylated protein kinase R (pPKR).

This table is submitted as separate excel file.

Supplementary Dataset 2: A table summarizing the percentage of immunopositive cells and the decay score after autolysis for 6 – 72 h and subsequent formalin fixation for 6 – 72 h in 10% neutral buffered formalin (NBF). The table displays infection status (1 = not infected; 2 = persistently infected with canine distemper virus (CDV; *Morbillivirus canis*), strain Onderstepoort (Ond)), formalin concentration, fixation time, the decay score and the number of immunopositive cells for the markers canine distemper virus nucleoprotein (CDV NP), interferon-stimulated gene 15 (ISG15), myxovirus resistance protein (Mx), interferon-β (IFNβ), and phosphorylated protein kinase R (pPKR). The decay score quantifies the degree of nuclear degeneration in stained tissue sections. Five high-power fields are evaluated, and cells are classified into five grades (1 = normal nuclear morphology to 5 = complete loss of recognizable cellular structures). The score is calculated as a weighted sum of the percentage of cells in each grade: Decay score = (% grade 1 × 1) + (% grade 2 × 2) + (% grade 3 × 3) + (% grade 4 × 4) + (% grade 5 × 5).

This table is submitted as separate excel file.
